# Effect of Food Proteins on Wheat Starch Pasting and Thermal Properties

**DOI:** 10.3390/foods14223865

**Published:** 2025-11-12

**Authors:** Andrés Gustavo Teobaldi, Esteban Josué Carrillo Parra, Gabriela Noel Barrera, Pablo Daniel Ribotta

**Affiliations:** 1 Instituto de Ciencia y Tecnología de los Alimentos Córdoba (ICYTAC-CONICET), Universidad Nacional de Córdoba, Av. Filloy S/N, Ciudad Universitaria, Córdoba X5000HUA, Argentina; ateobaldi@agro.unc.edu.ar (A.G.T.); ejocarrillo@mi.unc.edu.ar (E.J.C.P.); gbarrera@agro.unc.edu.ar (G.N.B.); 2 Departamento de Química Industrial y Aplicada, Facultad de Ciencias Exactas, Físicas y Naturales (FCEFyN), Universidad Nacional de Córdoba (UNC), Av. Vélez Sarsfield 1611, Córdoba X5000HUA, Argentina

**Keywords:** starch, pasting curves, modeling curves, Rapid Visco Analyzer, gelatinization

## Abstract

The objective of this study was to analyze the effect of different food proteins (wheat gluten, soy protein, whey protein, and ovalbumin), applied in different proportions, on the pasting and thermal properties of wheat starch using a Rapid Visco Analyzer (RVA) and Differential Scanning Calorimetry (DSC), enriching the RVA analysis with mathematical models for a deeper mechanistic understanding of the pasting behavior. Gluten and whey proteins significantly increased peak viscosity (up to +105% and +22%, respectively), while soy protein and ovalbumin decreased it (up to −16%). Conversely, the addition of all four proteins resulted in an increase in the starch pasting profile during the cooling period of the starch pastes (up to +95%). Additionally, the presence of all four proteins accelerated the starch paste formation process (0.2–0.9 min). Mathematical models showed that the addition of proteins accelerated both the viscosity decline phase (breakdown) immediately following the peak and the subsequent viscosity increase phase (setback), leading to the final viscosity. All proteins increased the gelatinization onset temperature, indicating restricted water availability for starch. Consequently, a decrease in gelatinization enthalpy was observed, most notably with ovalbumin (−28%) and whey protein (−24%). Conversely, the retrogradation characteristics showed no consistent pattern. These results offer valuable insights into managing the thermal stability of starch within diverse food applications (e.g., flour-based products) utilizing different protein sources.

## 1. Introduction

Starch and proteins are the main ingredients in food formulation, playing a crucial role in the nutritional profile and the physicochemical and rheological characteristics of final food products [1]. Most of the starches consumed by humans are thermally processed under conditions of humidity and shear, followed by cooling [2]. During hydrothermal treatment, starch granules absorb water and swell, causing the internal crystalline structures to break down in the gelatinization process [3]. After gelatinization, pasting follows, and further swelling of the granules together with the leaching of amylose molecules is produced. Pasting occurs at a higher temperature than gelatinization, causing the starch granules to be disrupted. This process leads to the formation of a starch paste, which can be evaluated from the system’s viscosity changes [3,4,5].

The Rapid Visco Analyzer (RVA) simulates the cooking process of cereal by subjecting a flour-water suspension to a cycle of heating, holding, cooling, and holding under constant shearing. During the heating phase, the starch reaches its gelatinization temperature, increasing paste viscosity until a maximum is reached. As the starch granules hydrate and swell, amylose and smaller amylopectin molecules leach out into the aqueous medium. In the subsequent hot holding stage, a drop in viscosity occurs. Several factors can affect this sudden decrease in viscosity, such as the swelling and rigidity of the starch granules, the content and/or molecular structure of amylopectin and amylose, and the lipids and proteins present in the starch. During the cooling phase, viscosity increases again as soluble amylose retrogrades, forming a gel that contains gelatinized starch granules [5,6].

Investigating pasting curves is a powerful tool for understanding starch’s technological and functional properties [7,8,9]. A deeper understanding of starch pasting analyses is crucial for analyzing gelatinization, determining food processing times, and selecting the appropriate starch type for specific food products. Therefore, employing a mathematical model may enhance our understanding of the relationship between starch components and their pasting behavior, as well as aid in the development of food products with desired characteristics. Additionally, a mathematical equation that can predict the rheological behavior of a product during production is invaluable for process design and quality control [10,11]. Moreover, modeling serves as a powerful tool for understanding the kinetics of gelatinization, which helps to establish the relationship between starch components and process time, as well as to predict the pasting behavior of the products [12]. While viscosity values can be obtained as a function of temperature during a pasting test using a Rapid Visco Analyzer, few studies have mathematically modeled the viscosity profile to elucidate the rates of processes such as gelatinization, breakdown, and retrogradation in starches [10,12,13,14,15].

In complex food matrices, the interaction between starch and proteins, particularly during thermal processing, is a critical determinant of the system’s final properties and functionality. At a molecular level, heat can induce the assembly of starch and protein chains into larger structures, which profoundly affects characteristics like texture and stability of the starch-based food matrices. For instance, studies utilizing ^13^C nuclear magnetic resonance have shown that glutenin can interact with amylose to form double helices, thereby reducing short-term starch retrogradation [16]. Therefore, a comprehensive understanding of these systems requires an examination of their multi-scale structures, from molecular ordering to morphology. Nevertheless, the precise mechanisms by which thermal processing and starch–protein interactions co-determine the final structural properties are still not fully elucidated [17]. While food proteins like gluten, soy protein, whey protein, and ovalbumin are of immense technological importance, the analysis of their interactions with starch has often been descriptive.

Pasting profiles, a key indicator of rheological behavior, have been interpreted without the application of mathematical models to isolate the underlying kinetic and structural changes. Consequently, the objective of this study was to employ mathematical modeling of pasting profiles, combined with thermal analysis, to quantitatively investigate and elucidate the impact of various food proteins on the pasting properties of wheat starch.

## 2. Materials and Methods

### 2.1. Samples

Commercial wheat starch (S) was used (moisture 11.0%, protein 0.6%, lipids 0.15% and ashes 0.5%, *w*/*w* dry basis, according to the manufacturer) and vital gluten (G) (humidity 10.0%, proteins 75.0%, lipids 1.5% and ashes 1.5% *w*/*w* dry basis, according to the manufacturer), both provided by Molinos Juan Semino S.A. (Santa Fe, Argentina). In turn, isolated soy protein (SP) (humidity 5.4%, proteins 91.2%, lipids 3.1%, ashes 1.2%, *w*/*w* dry basis, according to manufacturer), concentrated whey protein (WP) (moisture 5.0%, proteins 78.0%, lipids 9.0%, ash 2.3, *w*/*w* dry basis, according to manufacturer) and ovalbumin (ALB) (moisture 8.0%, proteins 80.0%, lipids 1.0%, ashes 6.0%, *w*/*w* dry basis, according to manufacturer) were provided by Pura Química SA (Córdoba, Argentina).

A total of 12 samples were prepared. Solid mixtures were prepared using commercial wheat starch and proteins (P) of different sources: gluten (G), soy (SP), whey (WP), and ovalbumin (ALB). The solids were mixed considering the particle size (400 mesh), and the homogeneity of the samples was ensured by stirring the solids overnight at a constant speed (oscillating stirrer). The percentages of proteins in the samples were: 10% (S/G90:10, S/SP90:10, S/WP90:10, S/ALB90:10), 20% (S/G80:20, S/SP80:20, S/WP80:20, S/ALB80:20), and 30% (S/G70:30, S/SP70:30, S/WP70:30, S/ALB70:30). In turn, the commercial wheat starch was used as a control.

### 2.2. Pasting Properties

The viscosity or pasting profiles of the samples were determined by a Rapid Visco Analyzer (RVA-4) using a general RVA pasting (Newport Scientific Pty. Ltd., Warriewood, Australia). The standard method for starch STD1 (Method 76-21; [3]) was used. Each sample (3.5 g, known humidity) was suspended in 25 g of water and shaken at 160 rpm and 50 °C for 1 min to achieve complete dispersion. Then the stirring speed was increased to 960 rpm and the temperature was increased to 95 °C. The heating rate was 9.4 °C/min. Next, the system temperature was kept constant at 95 °C for 2.5 min and then decreased to 50 °C at a cooling rate of 11.8 °C/min. The determinations were carried out in duplicate. The starch concentrations in the dispersions were 11.2% *w*/*w* (S/P90:10), 10.1% *w*/*w* (S/P80:20), and 9.6% *w*/*w* (S/P70:30). Additionally, a second set of samples was prepared with different concentrations of solids: pure starch in water (S90, S80, and S70), where the concentrations of starch were 11.2%, 10.1%, and 9.6%, respectively. This set of control samples was evaluated to analyze the effect of adding proteins at equal starch concentrations. During the pasting tests, the viscosity and temperature of the suspensions were recorded as a function of time, and, from the curves, the different parameters were obtained using the Thermocline software for Windows (V 3.17.3.509, Perten Instruments, Springfield, IL, USA). Each measurement was performed in duplicate.

### 2.3. Mathematical Modeling of Starch Pasting Behavior

**Figure 1:**
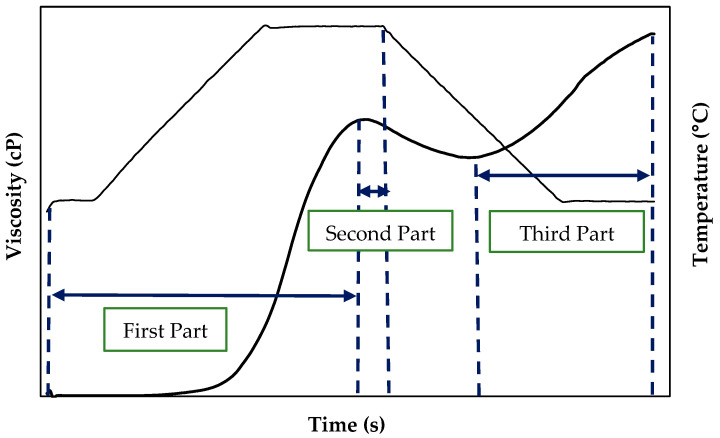
Schematic diagram of pasting curve parts used in each model.

The viscosity curves of the suspensions in water as a function of time obtained from the RVA were fitted to different equations using the StatGraphics software (Centurion XVII, Statpoint Technologies, Warrenton, VA, USA). For modeling, the pasting curves were divided into three sections following Palabiyik et al. (2017) [10] (Figure 1).

First part: this corresponds to the region where the temperature increased while the agitation was kept constant (960 rpm) until the peak of maximum viscosity. This section resembles a sigmoid and can be fitted to a logistic function. The experimental data were fitted to Equation (1) [10]:

(1) $$V_{1}\left(t\right)=\frac{PV\times t^{S}}{R^{S}+t^{S}}$$

where *PV* is the peak viscosity, related to the degree of swelling of the starch granules or the ability to bind free water; *t* is the pasting process time; *R* is the time in which the viscosity reached 50% of the peak viscosity value and allowed us to compare the resistance of the starch granules to heat and shear force; *S* is the starch coefficient, it represents the swelling rate of the granules and helped us to compare the internal (granule architecture, amylose-amylopectin ratio, damaged granules, compositions) and external (heating, shear rate) effects. Due to the nature of the model, it can be said that if *S* = 1, the initial penetration of water into the starch granule does not affect the subsequent entry of water molecules that may enter. If *S* < 1, the initial penetration of water into the starch granules negatively affects the entry of other water molecules, and their affinity for the latter decreases. If *S* > 1, the initial penetration of water into the starch granules positively affects the entry of subsequent water, and then these can easily enter the granules, thus increasing the granules’ swelling rate.

Second part: This region corresponds to the drop in viscosity after reaching the viscosity peak, in which the temperature (95 °C) and stirring speed (960 rpm) remained constant. The experimental data for this area of the pasting curve can be fitted using an exponential model (Equation (2)). The relationship between time and viscosity was expressed by Equation (2) [10]:

(2) $$V_{2}(t)=K^{nt}$$

where *V* represents the viscosity (*cP*), *K* and *n* are the model parameters, and *t* is the time of the pasting process.

Third part: in this curve region, the temperature decreased (from 95 °C to 50 °C) and the stirring speed remained constant (960 rpm). This curve area began at the minimum viscosity after the drop of the viscosity peak (average viscosity −VM−) until the end of the test (final viscosity −FV−). The experimental data were fitted to an Arrhenius equation (Equation (3)) [10] to determine the relationship between temperature and viscosity.

(3) $$V_{3}(t)={A_{0}}^{\left(\frac{E_{a}}{RT}\right)}$$

where *A*_0_ is the constant parameter of the model, *Ea* is the activation energy, *R* is the gas constant, and *T* is the temperature (in Kelvin).

### 2.4. Differential Scanning Calorimetry (DSC)

Samples (~10 mg) and water (~30 μL) were weighed in an aluminum pan (100 μL). The aluminum pans were sealed and equilibrated at room temperature for at least 12 h before heating analysis to equilibrate the solids-water mixture. Thermal studies were performed with a DSC823e Calorimeter, and thermograms were evaluated by STARe Default DB V9.00 software (Mettler Toledo, Greifensee, Switzerland). The DSC analyzer was calibrated using indium, and an empty aluminum pan was used as a reference. The samples were held at 25 °C for 5 min and heated from 25 to 110 °C at 5 °C/min. Onset temperature (T_O_ (°C)), peak width at half height (PW_g_ (°C)), and change in enthalpy referred to gelatinization (ΔH_g_ (J/g starch)) were determined. All measurements were carried out in triplicate.

For this analysis, a set of control samples with different solids concentrations was used: starch in water (S90, S80, and S70), where starch concentrations were 23.1%, 21.1%, and 18.9%. This set of samples was analyzed to compare the effect of protein addition while keeping the amount of added starch constant (but without protein).

To evaluate the amylopectin retrogradation process, the pans containing the gelatinized samples (after the heating process) were stored at 4 °C for 21 days. The pans were then heated again in the calorimeter from 25 °C to 110 °C at a heating rate of 5 °C/min. The following parameters were calculated from the thermograms obtained: retrogradation onset temperature (T_Or_), peak width (PW_r_), and enthalpy change in starch retrogradation (ΔH_r_). Measurements were made in triplicate.

### 2.5. Statistical Analysis

Data were statistically treated using analysis of variance (ANOVA). The means were compared using an LSD Fisher test at a significance level of 0.05. The relationship between the variables was studied by Principal Component Analysis (PCA). INFOSTAT statistical software v2020 (Facultad de Ciencias Agropecuarias, UNC, Córdoba, Argentina) was used for statistical analysis.

## 3. Results and Discussion

### 3.1. Effect of Proteins on the Pasting Properties of Starch

**Figure 2:**
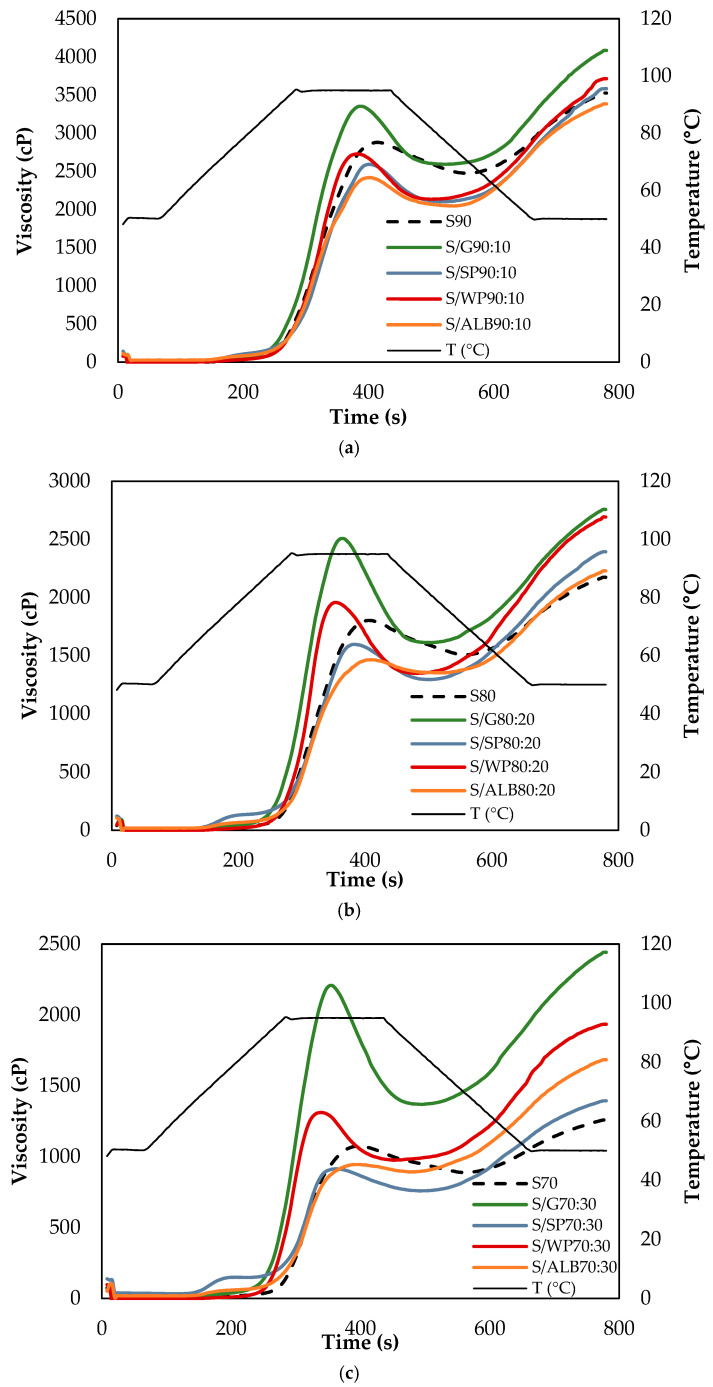
Effect of proteins at (**a**) 10%, (**b**) 20% and (**c**) 30% on the apparent viscosity plots for starch pasting. S: starch, G: gluten, SP: soy protein, WP: whey protein, ALB: ovalbumin.

The pasting curves of the samples are presented in Figure 2, and the pasting parameters are summarized in Table 1. Based on the absolute mean values of the RVA parameters, the percentages of increase (+) or decrease (−) for these parameters were calculated (Table 2). These percentages, associated with protein addition, were determined by comparing the values obtained for the dispersions S90 with S/P90:10, S80 with S/P80:20, and S70 with S/P70:30 for each protein studied.

The pasting properties of wheat starch were distinctly altered by protein type. During the heating phase, gluten and whey proteins significantly increased (*p* < 0.05) PV and BD, whereas ovalbumin decreased; soy protein caused a slight reduction in PV. During the cooling phase, all proteins significantly increased (*p* < 0.05) FV and SB, and the most pronounced effect was on gluten and whey protein. The time to reach peak viscosity was shortened by gluten, soy, and whey proteins, but remained unchanged with ovalbumin (Table 1 and Table 2).

The results suggest an interaction between the starch granules and the leached amylose molecules with the proteins and/or a competition between the starch and the proteins for the available water. The reasons for the effect of proteins on pasting properties can be considered from the following three aspects: (a) starch and proteins can develop complexes with different structures and molecular weights; (b) the water adsorption capacity of proteins can modify the available water for the starch granules; and (c) the thermal phase transitions of proteins (denaturalization, conformational changes) can affect energy transmission during gelatinization [17].

The peak viscosity of a pure-starch dispersion is governed by the amount of leached amylose, the swelling of the starch granules, and the competition for free water between the leached amylose molecules and the swelled starch granules. In starch–gluten systems, there may be competition between starch and gluten for available water, which increases the effective concentration of starch and, consequently, leads to an increase in peak viscosity. Furthermore, during heating, gluten proteins increase their water absorption capacity, resulting in a rise in viscosity [18]. Due to the formation of gluten aggregates, the free volume of the aqueous phase and the free energy of starch dispersion decrease. In this system, the mobility of the particles is restricted, which tends to increase the viscosity. Interactions between starch granules and leached molecules with gluten proteins can lead to the formation of a cross-linked structure, resulting in increased viscosity. Conversely, the reduction in viscosity that occurs with the presence of soy proteins and ovalbumin may be due to their interaction with starch granules and leached amylose molecules. This interaction can form a layer of proteins around the starch granules, thereby creating a barrier that prevents water from entering the granules and their swelling [19]. This drop in peak viscosity can also be attributed to the fact that soy protein and ovalbumin interact more strongly with water due to their conformational arrangements, limiting the availability and mobility of water in the system.

Breakdown viscosity reflects the rupture of hydrated and gelatinized starch granules under shear. An increase in breakdown can be associated with higher disintegration of the granules and the alignment of the amylose chains with the flow direction. Furthermore, it may be related to the granules’ lower resistance to shear force, leading to greater rupture [2,5]. Generally, an increase in the breakdown of starch granules is associated with higher peak viscosities reached in starch systems because the granules present greater swelling and therefore are more easily disintegrable, which agrees with PV values for gluten and ovalbumin. By contrast, the addition of soy and whey proteins caused a decrease in BD values. This suggests that these proteins could partially prevent the swelling of starch granules, thus leading to the preservation of granular integrity during shearing, causing less disintegration [2,20].

The final viscosity of the starch pastes is reached after cooling at the end of the RVA test. Both the FV and the SB depend on the degree of leached starch molecules’ interactions (amylose mainly), and the content of amylose molecules leached from the granules during gelatinization. The increases in viscosity due to the addition of proteins can be attributed to their denaturation, aggregation, and gelation as a result of thermal treatments [21]. The denaturation of globular proteins (gluten, soy protein, whey protein, and ovalbumin) results in the unfolding and exposure of different amino acid residues. This leads to changes in the functional properties of proteins, such as gel formation, protein aggregation, and dispersion in solution [22,23]. The interior of globular proteins contains mainly hydrophobic residues, which facilitate the formation of greater protein–protein interactions and interactions between proteins with the hydrophobic components of the matrix [23,24]. Aggregation is due to hydrophobic, electrostatic, and hydrogen bonding interactions, as well as covalent bonds, depending on the location and exposure of the protein residues. In a starch–protein mixture, the unfolding and subsequent aggregation of proteins can influence the functionality of starch. While thermally induced protein aggregation can lead to precipitation and loss of protein functionality, the denaturation of some globular proteins results in gel formation [24]. The formation of a protein gel may impact the mobility of water within the starch–protein matrix. The swelling of starch granules and the stability of the starch–protein matrix during storage are dependent on the mobility of water in the system [25]. Consequently, the protein gelling capacity and the microstructure (strength, uniformity, surface charge) of the resulting gel networks could affect the performance of the starch granules [26].

### 3.2. Modeling of Pasting Curves

To further explore the effects of proteins on the viscous and thermal properties of starch dispersions and to predict the behavior of protein-starch matrices, mathematical models were applied to viscosity data over time. These models helped analyze aspects such as how quickly the viscosity peak is reached, which contributes to understanding the gelatinization kinetics of starch granules (first part of the curve). Additionally, the models provided information on the rate at which viscosity decreased after reaching the maximum peak (second part of the curve) and the rate of viscosity increases during the cooling phase of the starch paste in the gelation process (third part of the curve).

In the initial part of the RVA curves, the parameters *PV*, *R*, and *S* (Equation (1)) were obtained (Table 3). The viscosity profiles of all samples highly correlated with the model (*R*^2^ values greater than 0.95). The observed trends in *PV* for the experimental data and those calculated by the model were similar. The *PV* values predicted by the model showed an increase (*p* < 0.05) with the addition of 20% and 30% of gluten, soy, and whey proteins. However, a decrease (*p* < 0.05) in *PV* was observed with the addition of ovalbumin. The parameter *R* decreased significantly (*p* < 0.05) with the addition of gluten and whey proteins, indicating a faster pasting rate, which was consistent with a reduction in the peak time (Table 1). In contrast, soy protein and ovalbumin had no significant effect on *R*. The *S* values were greater than 1 for all protein aggregates, suggesting that the initial water absorption into the starch granules facilitates the subsequent entry of other water molecules. The addition of 10% and 20% gluten, as well as all proportions of whey protein, increased (*p* < 0.05) the absolute value of *S*. The increased *S* with gluten and whey proteins relates to the lower *R* values and higher *PV* observed. Conversely, a significant decrease in *S* was observed at 10% and 20% soy protein levels, as well as at 30% ovalbumin. These results suggest that soy protein and ovalbumin interact more strongly with water than gluten and whey protein, thereby restricting water availability and thus granule swelling.

In the second part of the viscosity profiles, the curves were fitted to an exponential model, and the obtained parameters (*k* and *n*) (Equation (2)) are detailed in Table 3. The fits for all curves presented R^2^ values greater than 0.93. The value of *n* is related to the rate of viscosity decay under the test conditions: the higher the value of *n*, the greater the rate of viscosity decay after reaching the peak. The addition of gluten and whey proteins resulted in more abrupt drops (*p* < 0.05) in the viscosity of the starch paste at 95 °C. This revealed that the higher the percentage of protein addition, the greater the decay rate. However, the addition of ovalbumin caused the opposite effect, suggesting that starch granules fragmented more rapidly in the presence of gluten and whey protein. These trends are related to peak viscosity values. The higher the peak viscosity, the greater the swelling of the granules, and therefore, the greater their fragility under the shear force applied during the test. This led to larger and more rapid drops in viscosity after reaching the peak viscosity. These results were in agreement with the BD values (Table 1) since a faster viscosity drop may be related to a greater sensitivity of the granules under shear forces and high temperatures.

In the fitting equation for the third region of the curve (Equation (3)), the *E*_a_ parameter can be interpreted as a retrogradation rate, due to the structuring of the amylose chains leached from the starch granules during gelatinization, which form rigid structures by interaction with the amylopectin chains of the swollen granules [10]. It could be observed that the *E_a_* values increased with the addition of the four proteins. The presence of proteins can limit the availability of water; therefore, there could be greater proximity between the leached amylose chains to interact with each other and thus form the network.

### 3.3. Differential Scanning Calorimetry (DSC)

DSC parameters of gelatinization and retrogradation processes are presented in Table 4. Based on the absolute mean values of gelatinization and retrogradation enthalpy, the percentages of increase (+) or decrease (−) for this parameter were calculated (Table 5). These percentages, associated with protein addition, were determined by comparing the values obtained for the dispersions S90 with S/P90:10, S80 with S/P80:20, and S70 with S/P70:30 for each protein studied. By contrast, the differences between the onset temperatures (ΔT_O_ and ΔT_Or_) and the peak width (ΔPW_g_ and ΔPW_r_) of gelatinization and retrogradation were calculated, and samples were compared with their respective controls.

#### 3.3.1. Gelatinization

As a general trend, a decrease in the gelatinization enthalpy value was observed with the addition of protein. Whey protein and ovalbumin caused the greatest decrease in this parameter. Regarding the gelatinization onset temperature, an increase in this parameter was detected as a result of the addition of the proteins studied. The greatest values were seen with the addition of ovalbumin. Finally, no trend was observed regarding the effect of proteins on the peak width of starch gelatinization.

According to Ratnayake and Jackson [27], starch gelatinization is a gradual process with three main stages: (1) starch granules absorb water, increasing the mobility of starch polymers, especially amylose, in the amorphous regions; (2) these polymers rearrange and form new intermolecular bonds; and (3) with more heat, the polymers become even more mobile, lose their bonds, and the granular structure breaks down. During this process, energy melts the crystalline structures and helps create new molecular bonds, known as rearrangement. This theory suggests that the decrease in the enthalpy of gelatinization in the presence of gluten, whey, and ovalbumin proteins may be related to the formation of different interactions during the second gelatinization phase, which leads to polymer conformations associated with lower energies. In this study, whey proteins and ovalbumin caused greater decreases in this thermal parameter, indicating that they favor the formation of polymer conformations with lower energies. This implies that the proteins could interact with starch molecules through hydrogen bonding interactions. Biliaderis et al. (1986) [28] proposed a similar theory considering starch gelatinization as a three-stage process. One of these involves the recrystallization of starch polymers. Since the addition of gluten proteins, whey protein, and ovalbumin decreased gelatinization enthalpy, the recrystallization phase is predominant in the presence of these proteins. Furthermore, aggregates of these proteins may interact with the surface of starch granules [19], creating a protein barrier and potentially interfering with water diffusion into the starch granules and leading to a reduction in the fraction of gelatinized granules. The addition of all proteins increased the onset temperature of gelatinization, suggesting a delay in the disorganization of the amorphous regions of starch during gelatinization. This delay can be attributed to limited water availability for starch gelatinization, which resulted from proteins’ hydration capacity and a decrease in the rate of water migration from proteins to starch [29]. To sum up, the increase in the onset gelatinization temperature as gelatinization enthalpy is reduced could be related to the water availability in the systems, which is affected by the water holding capacity of each polymer (starch and proteins), as well as the interfering effect of proteins on water diffusion into the starch granules. Consequently, reductions in water availability in the systems could lead to incomplete granule swelling and a decrease in the starch crystalline fraction that can melt [30,31].

#### 3.3.2. Retrogradation

As general trends, a shift in the retrogradation endotherm to higher temperatures (especially for the 10% aggregate of the four proteins studied) and a narrowing of the endotherm width were observed. Regarding the retrogradation enthalpy, the values did not show a clear trend.

The effects of proteins on amylopectin retrogradation can be explained by water mobility, hydrogen bonds, electrostatic and hydrophobic interactions, and covalent bonds [32]. Protein–protein, starch–protein, and starch–protein-water interactions affected the retrogradation rate of amylopectin. Hydrogen-bonding interactions are known to occur between molecular chains during reassociation. These interactions also occurred between proteins, contributing to their aggregation, which limited protein-water interactions and, consequently, favored the water mobility in the starch systems, promoting amylopectin retrogradation.

The hydrogen-bonding interactions and covalent bonds formed during this process between starch chains and proteins could favor amylopectin retrogradation and lead to the formation of more stable and organized crystals. This could explain the increase in the retrogradation onset temperature that was observed when adding different proteins to starch.

### 3.4. Principal Component Analysis (PCA)

The relationship between pasting and thermal properties of starch–protein systems was analyzed using principal component analysis. PCA was performed for all samples at the same time to evaluate the influence of parameters on the samples’ variability. The PCA scatter-plot represented 77.2% of the samples’ total variability (Figure S1). The PC1 projection explained 60.5% of the samples’ variability and was defined by pasting parameters (A_0_, PV, FV, PT, BD, and SB) and one DSC parameter (T_O_). The contribution of the parameters to the samples’ variability was similar. Samples were divided along PC1 into two groups according to their protein content: one group of samples with higher protein content (20 and 30%), and another group of samples with lower protein content (10%, except for S/G80:20 and S/G70:30).

The PC2 projection explained 16.7% of the samples’ variability, and the DSC parameters ΔH_g_, PW_g_, T_Or_, and PW_r_ were the most influential variables in the samples’ variability. PC2 separated samples into two groups based on protein type: one group consisting of samples with gluten (except S/G80:20), whey, and ovalbumin, and another group where only soy protein was found.

As a consequence of the singular behavior of S/G80:20 and S/G70:30, each group of samples (samples with the addition of 10% protein, samples with the addition of 20% protein, and samples with the addition of 30% protein) was analyzed separately by PCA, considering the same set of variables.

For samples with 10% protein added, the PCA scatter-plot represented 80.9% of the total samples’ variability (Figure S2). The PC1 projection explained 66.2% of the samples’ variability and was defined by K, A_0_, E_a_, PV, FV, and BD, which showed the highest contributions to the variability. PC1 separated the samples with a 10% protein addition into two groups: one group consisting of samples with soy protein, whey, and ovalbumin, and another group where only gluten was found.

The PCA scatter-plot for samples with 20% added protein represented 83.0% of the total sample variability (Figure S3). The PC1 projection accounted for 59.3% of the sample variability. It was defined by the parameters K, A_0_, PV, FV, BD, PT, ΔH_g_, T_O_, PW_g_, T_Or_, and PW_r_, which showed the greatest contributions to the variability. PC1 separated the samples with 20% added protein into two groups: one group consisting of samples with soy protein, whey, and ovalbumin, and another group where only gluten was found.

The PCA scatter-plot for samples with 30% of added protein represented 88.2% of the total sample variability (Figure S4). The PC1 projection accounted for 62.7% of the sample variability and was defined by K, A_0_, PV, FV, SB, BD, and PT, which showed the highest contributions to the variability. PC1 separated the samples with 30% added protein into two groups: one group consisting of samples with soy protein, whey, and ovalbumin, and another group where only gluten was found.

## 4. Conclusions

This study systematically investigated the impact of proteins from diverse origins (gluten, soy, whey, and ovalbumin) on the pasting and phase transition properties of wheat starch. The pasting profiles were highly dependent on protein type and concentration. Gluten and whey proteins were observed to increase peak viscosity, while soy protein and ovalbumin suppressed it. These divergent effects are attributed to competitive water binding and specific interactions between the proteins, starch granules, and leached amylose. Mathematical models were successfully applied to the pasting curves, providing deeper mechanistic insight, as well as a novel and quantitative analysis of the gelatinization kinetics, shear breakdown, and retrogradation behavior. The model parameters robustly complemented the empirical RVA data.

Understanding the rheological and thermal behavior of starch–protein slurries is critical for designing food textures and predicting product performance. DSC further confirmed that all proteins restricted water availability, as evidenced by a significant increase in gelatinization onset temperature and a decrease in gelatinization enthalpy. This restricted the full swelling of starch granules and the melting of crystallites in the presence of food proteins. The results of the statistical analysis revealed that PCA effectively separated the starch–protein systems based on the protein type. Remarkably, the starch–gluten system demonstrated a pronounced viscosity profile, a distinction that was robustly confirmed by the PCA model.

This research provides a fundamental understanding of how specific proteins modulate starch functionality. The use of mathematical models in pasting curves proved to be a novel tool for studying the pasting properties of starch. Further studies are needed to relate these results to the pasting and thermal properties of starch-based food systems containing starch with the different proteins studied. The findings from these analyses serve as a fundamental basis for studying complex food matrices like bakery products, in which components such as egg albumin and dairy-based whey proteins are commonly incorporated.

## Figures and Tables

**Table 1:** Effect of protein incorporation on wheat starch pasting parameters. Values followed by different letters in the same column are significantly different (*p* < 0.05). S: starch, G: gluten, SP: soy protein, WP: whey protein, ALB: ovalbumin.

Sample	PV (cP)	BD (cP)	FV (cP)	SB (cP)	Pt (min)	PT (°C)
S90	2880 ± 2 ^a^	449 ± 59 ^a^	3527 ± 37 ^a^	1096 ± 20 ^a^	6.9 ± 0.0 ^b^	86.8 ± 0.6 ^a^
S/G90:10	3357 ± 58 ^b^	764 ± 57 ^b^	4085 ± 64 ^b^	1492 ± 62 ^b^	6.5 ± 0.1 ^a^	85.2 ± 0.6 ^a^
S90	2880 ± 2 ^b^	449 ± 59 ^a^	3527 ± 37 ^a^	1096 ± 20 ^a^	6.9 ± 0.0 ^b^	86.8 ± 0.6 ^a^
S/SP90:10	2594 ± 23 ^a^	492 ± 69 ^a^	3583 ± 37 ^a^	1481 ± 82 ^b^	6.7 ± 0.0 ^a^	89.2 ± 0.5 ^b^
S90	2880 ± 2 ^b^	449 ± 59 ^a^	3527 ± 37 ^a^	1096 ± 20 ^a^	6.9 ± 0.0 ^b^	86.8 ± 0.6 ^a^
S/WP90:10	2730 ± 12 ^a^	595 ± 12 ^a^	3715 ± 4 ^b^	1580 ± 28 ^b^	6.4 ± 0.0 ^a^	87.6 ± 0.6 ^a^
S90	2880 ± 2 ^b^	449 ± 59 ^a^	3527 ± 37 ^b^	1096 ± 20 ^a^	6.9 ± 0.0 ^b^	86.8 ± 0.6 ^a^
S/ALB90:10	2420 ± 4 ^a^	373 ± 23 ^a^	3385 ± 2 ^a^	1338 ± 28 ^b^	6.7 ± 0.0 ^a^	88.4 ± 0.6 ^a^
S80	1803 ± 30 ^a^	294 ± 13 ^a^	2175 ± 43 ^a^	666 ± 25 ^a^	6.7 ± 0.0 ^b^	90.5 ± 0.0 ^b^
S/G80:20	2509 ± 9 ^b^	896 ± 20 ^b^	2758 ± 8 ^b^	1146 ± 19 ^b^	6.1 ± 0.0 ^a^	86.3 ± 0.1 ^a^
S80	1803 ± 30 ^b^	294 ± 13 ^a^	2175 ± 43 ^a^	666 ± 25 ^a^	6.7 ± 0.0 ^b^	90.5 ± 0.0 ^a^
S/SP80:20	1598 ± 45 ^a^	303 ± 3 ^a^	2394 ± 2 ^b^	1099 ± 40 ^b^	6.4 ± 0.0 ^a^	92.0 ± 0.1 ^b^
S80	1803 ± 30 ^a^	294 ± 13 ^a^	2175 ± 43 ^a^	666 ± 25 ^a^	6.7 ± 0.0 ^b^	90.5 ± 0.0 ^a^
S/WP80:20	1958 ± 40 ^b^	609 ± 17 ^b^	2693 ± 22 ^b^	1344 ± 1 ^b^	5.9 ± 0.0 ^a^	89.3 ± 0.6 ^a^
S80	1803 ± 30 ^b^	294 ± 13 ^b^	2175 ± 43 ^a^	666 ± 25 ^a^	6.7 ± 0.0 ^a^	90.5 ± 0.0 ^a^
S/ALB80:20	1468 ± 62 ^a^	116 ± 17 ^a^	2229 ± 110 ^a^	878 ± 66 ^a^	6.9 ± 0.1 ^a^	91.7 ± 0.6 ^a^
S70	1076 ± 33 ^a^	189 ± 5 ^a^	1259 ± 4 ^a^	371 ± 34 ^a^	6.6 ± 0.1 ^b^	93.3 ± 0.5 ^b^
S/G70:30	2208 ± 15 ^b^	839 ± 18 ^b^	2442 ± 0 ^b^	1073 ± 3 ^b^	5.9 ± 0.0 ^a^	87.2 ± 0.1 ^a^
S70	1076 ± 33 ^b^	189 ± 5 ^a^	1259 ± 4 ^a^	371 ± 34 ^a^	6.6 ± 0.1 ^a^	93.3 ± 0.5 ^a^
S/SP70:30	916 ± 16 ^a^	158 ± 21 ^a^	1394 ± 7 ^b^	636 ± 2 ^b^	6.0 ± 0.1 ^a^	94.1 ± 0.6 ^a^
S70	1076 ± 33 ^a^	189 ± 5 ^a^	1259 ± 4 ^a^	371 ± 34 ^a^	6.6 ± 0.1 ^b^	93.3 ± 0.5 ^b^
S/WP70:30	1312 ± 35 ^b^	337 ± 12 ^b^	1934 ± 13 ^b^	959 ± 10 ^b^	5.7 ± 0.0 ^a^	88.4 ± 0.6 ^a^
S70	1076 ± 33 ^a^	189 ± 5 ^b^	1259 ± 4 ^a^	371 ± 34 ^a^	6.6 ± 0.1 ^a^	93.3 ± 0.5 ^a^
S/ALB70:30	945 ± 54 ^a^	53 ± 4 ^a^	1684 ± 87 ^b^	792 ± 36 ^b^	6.5 ± 0.0 ^a^	93.3 ± 0.5 ^a^

**Table 2:** Effect of protein addition on the pasting properties of wheat starch. Percentages of increase (+) or decrease (−) of the parameters obtained in RVA (PV, BD, FV, and SB). Ns: no significant differences. S: starch, G: gluten, SP: soy protein, WP: whey protein, ALB: ovalbumin.

[Protein]	Effect	Protein	PV (%)	BD (%)	FV (%)	SB (%)
**10%**	Protein addition	G	+17	+70	+16	+36
		SP	−10	Ns	Ns	+35
		WP	−5	Ns	+5	+44
		ALB	−16	Ns	−4	+22
**20%**	Protein addition	G	+39	+185	+27	+72
		SP	−11	+3	+10	+65
		WP	+9	+107	+24	+102
		ALB	−19	−61	Ns	Ns
**30%**	Protein addition	G	+105	+375	+94	+189
		SP	−15	Ns	+11	+71
		WP	+22	+79	+54	+158
		ALB	Ns	−72	+34	+113

**Table 3:** Fitting parameters determined from experimental data of RVA curves for protein incorporation. Values followed by different letters in the same column are significantly different (*p* < 0.05). S: starch, G: gluten, SP: soy protein, WP: whey protein, ALB: ovalbumin.

Sample	1st Part				2nd Part			3rd Part		
	PVm (cP)	R (min)	S	R^2^	K (cP)	n×100	R^2^	A_0_	E_a_ (J/mol)	R^2^
S90	3119 ± 16 ^a^	5.42 ± 0.06 ^a^	11.3 ± 0.1 ^a^	0.999	3993 ± 80 ^a^	0.08 ± 0.00 ^a^	0.937	1615.2 ± 48.3 ^a^	241.1 ± 17.8 ^a^	0.979
S/G90:10	3717 ± 98 ^b^	5.27 ± 0.04 ^a^	12.0 ± 0.2 ^b^	0.999	9081 ± 400 ^b^	0.25 ± 0.01 ^b^	0.970	1643.5 ± 40.9 ^a^	274.2 ± 17.6 ^a^	0.951
S90	3119 ± 16 ^b^	5.42 ± 0.06 ^a^	11.3 ± 0.1 ^b^	0.999	3993 ± 80 ^a^	0.08 ± 0.00 ^a^	0.937	1615.2 ± 48.3 ^b^	241.1 ± 17.8 ^a^	0.979
S/SP90:10	3027 ± 18 ^a^	5.55 ± 0.05 ^a^	10.5 ± 0.0 ^a^	0.997	5646 ± 1095 ^a^	0.19 ± 0.05 ^a^	0.948	1206.6 ± 78.5 ^a^	338.1 ± 30.5 ^b^	0.969
S90	3119 ± 16 ^a^	5.42 ± 0.06 ^a^	11.3 ± 0.1 ^a^	0.999	3993 ± 80 ^a^	0.08 ± 0.00 ^a^	0.937	1615.2 ± 48.3 ^b^	241.1 ± 17.8 ^a^	0.979
S/WP90:10	3118 ± 13 ^a^	5.37 ± 0.00 ^a^	13.4 ± 0.0 ^b^	0.999	7435 ± 298 ^b^	0.26 ± 0.01 ^b^	0.963	1262.7 ± 33.1 ^a^	333.5 ± 10.1 ^b^	0.972
S90	3119 ± 16 ^b^	5.42 ± 0.06 ^a^	11.3 ± 0.1 ^a^	0.999	3993 ± 80 ^a^	0.08 ± 0.00 ^a^	0.937	1615.2 ± 48.3 ^b^	241.1 ± 17.8 ^a^	0.979
S/ALB90:10	2667 ± 10 ^a^	5.37 ± 0.02 ^a^	11.2 ± 0.2 ^a^	0.998	4932 ± 396 ^a^	0.18 ± 0.02 ^b^	0.958	1030.7 ± 40.8 ^a^	409.1 ± 16.6 ^b^	0.991
S80	1926 ± 37 ^a^	5.39 ± 0.00 ^b^	12.9 ± 0.0 ^a^	0.999	2716 ± 159 ^a^	0.10 ± 0.01 ^a^	0.932	937.2 ± 25.3 ^a^	267.6 ± 7.5 ^a^	0.977
S/G80:20	2818 ± 11 ^b^	5.14 ± 0.01 ^a^	13.9 ± 0.1 ^b^	0.999	14,048 ± 1320 ^b^	0.46 ± 0.02 ^b^	0.989	924.6 ± 15.8 ^a^	357.8 ± 7.1 ^b^	0.993
S80	1926 ± 37 ^a^	5.39 ± 0.00 ^a^	12.9 ± 0.0 ^b^	0.999	2716 ± 159 ^a^	0.10 ± 0.01 ^a^	0.932	937.2 ± 25.3 ^b^	267.6 ± 7.5 ^a^	0.977
S/SP80:20	2480 ± 307 ^a^	5.78 ± 0.23 ^a^	8.5 ± 0.7 ^a^	0.989	3338 ± 112 ^b^	0.19 ± 0.00 ^b^	0.955	660.7 ± 30.7 ^a^	437.0 ± 10.8 ^b^	0.995
S80	1926 ± 37 ^a^	5.39 ± 0.00 ^b^	12.9 ± 0.0 ^a^	0.999	2716 ± 159 ^a^	0.10 ± 0.01 ^a^	0.932	937.2 ± 25.3 ^b^	267.6 ± 7.5 ^a^	0.977
S/WP80:20	2279 ± 10 ^b^	5.19 ± 0.03 ^a^	17.3 ± 0.5 ^b^	0.998	8603 ± 287 ^b^	0.41 ± 0.00 ^b^	0.977	645.0 ± 13.3 ^a^	494.8 ± 3.9 ^b^	0.987
S80	1926 ± 37 ^b^	5.39 ± 0.00 ^a^	12.9 ± 0.0 ^a^	0.999	2716 ± 159 ^a^	0.10 ± 0.01 ^a^	0.932	937.2 ± 25.3 ^b^	267.6 ± 7.5 ^a^	0.977
S/ALB80:20	1539 ± 66 ^a^	5.30 ± 0.04 ^a^	13.0 ± 0.2 ^a^	0.996	2162 ± 121 ^a^	0.09 ± 0.00 ^a^	0.983	766.9 ± 21.0 ^a^	347.2 ± 9.9 ^b^	0.973
S70	1108 ± 38 ^a^	5.31 ± 0.07 ^b^	17.0 ± 0.9 ^a^	0.999	1794 ± 104 ^a^	0.13 ± 0.02 ^a^	0.968	545.8 ± 47.6 ^a^	272.2 ± 31.0 ^a^	0.985
S/G70:30	2486 ± 28 ^b^	5.07 ± 0.01 ^a^	14.8 ± 0.0 ^a^	0.999	13,785 ± 72 ^b^	0.51 ± 0.00 ^b^	0.991	732.0 ± 1.3 ^b^	437.0 ± 10.8 ^b^	0.989
S70	1108 ± 38 ^a^	5.31 ± 0.07 ^a^	17.0 ± 0.9 ^b^	0.999	1794 ± 104 ^a^	0.13 ± 0.02 ^a^	0.968	545.8 ± 47.6 ^b^	272.2 ± 31.0 ^a^	0.985
S/SP70:30	6726 ± 187 ^b^	9.21 ± 0.47 ^b^	4.9 ± 0.3 ^a^	0.955	1847 ± 138 ^a^	0.19 ± 0.02 ^a^	0.986	371.0 ± 3.0 ^a^	470.4 ± 0.9 ^b^	0.991
S70	1108 ± 38 ^a^	5.31 ± 0.07 ^b^	17.0 ± 0.9 ^a^	0.999	1794 ± 104 ^a^	0.13 ± 0.02 ^a^	0.968	545.8 ± 47.6 ^a^	272.2 ± 31.0 ^a^	0.985
S/WP70:30	1420 ± 38 ^b^	4.89 ± 0.01 ^a^	20.3 ± 0.0 ^b^	0.999	4377 ± 255 ^b^	0.35 ± 0.01 ^b^	0.975	500.4 ± 20.2 ^a^	465.7 ± 9.2 ^b^	0.976
S70	1108 ± 38 ^a^	5.31 ± 0.07 ^a^	17.0 ± 0.9 ^b^	0.999	1794 ± 104 ^b^	0.13 ± 0.02 ^a^	0.968	545.8 ± 47.6 ^a^	272.2 ± 31.0 ^a^	0.985
S/ALB70:30	1033 ± 67 ^a^	5.25 ± 0.03 ^a^	13.3 ± 0.2 ^a^	0.990	1214 ± 112 ^a^	0.06 ± 0.00 ^a^	0.944	479.1 ± 25.9 ^a^	427.4 ± 1.5 ^b^	0.993

**Table 4:** Effect of protein incorporation on wheat starch DSC parameters. Values followed by different letters in the same column are significantly different (*p* < 0.05). S: starch, G: gluten, SP: soy protein, WP: whey protein, ALB: ovalbumin.

Sample	ΔH_g_ (J/g)	T_O_ (°C)	PW_g_ (°C)	ΔH_r_ (J/g)	T_Or_ (°C)	PW_r_ (°C)
S90	7.16 ± 0.09 ^b^	55.4 ± 0.7 ^a^	7.3 ± 0.1 ^a^	2.41 ± 0.47 ^a^	37.1 ± 0.3 ^a^	12.0 ± 0.6 ^a^
S/G90:10	6.39 ± 0.12 ^a^	56.3 ± 0.6 ^a^	7.2 ± 0.1 ^a^	2.89 ± 0.54 ^a^	42.2 ± 2.1 ^a^	12.7 ± 0.7 ^a^
S90	7.16 ± 0.09 ^a^	55.4 ± 0.7 ^a^	7.3 ± 0.1 ^a^	2.41 ± 0.47 ^a^	37.1 ± 0.3 ^a^	12.0 ± 0.6 ^a^
S/SP90:10	7.88 ± 0.27 ^a^	56.5 ± 0.2 ^a^	7.5 ± 0.3 ^a^	2.07 ± 0.08 ^a^	41.5 ± 0.4 ^b^	12.7 ± 0.3 ^a^
S90	7.16 ± 0.09 ^a^	55.4 ± 0.7 ^a^	7.3 ± 0.1 ^b^	2.41 ± 0.47 ^a^	37.1 ± 0.3 ^a^	12.0 ± 0.6 ^a^
S/WP90:10	6.56 ± 0.43 ^a^	57.0 ± 0.0 ^a^	6.2 ± 0.0 ^a^	2.24 ± 0.08 ^a^	43.7 ± 0.1 ^b^	10.1 ± 0.4 ^a^
S90	7.16 ± 0.09 ^b^	55.4 ± 0.7 ^a^	7.3 ± 0.1 ^b^	2.41 ± 0.47 ^a^	37.1 ± 0.3 ^a^	12.0 ± 0.6 ^b^
S/ALB90:10	5.98 ± 0.02 ^a^	57.8 ± 0.6 ^b^	6.5 ± 0.1 ^a^	1.98 ± 0.06 ^a^	44.7 ± 0.6 ^b^	9.5 ± 0.0 ^a^
S80	7.88 ± 0.06 ^b^	55.3 ± 0.4 ^a^	7.0 ± 0.2 ^a^	2.08 ± 0.14 ^a^	41.6 ± 0.5 ^a^	12.4 ± 0.2 ^b^
S/G80:20	6.75 ± 0.20 ^a^	56.3 ± 0.3 ^b^	7.3 ± 0.2 ^a^	1.93 ± 0.21 ^a^	40.6 ± 0.1 ^a^	10.7 ± 0.2 ^a^
S80	7.88 ± 0.06 ^b^	55.3 ± 0.4 ^a^	7.0 ± 0.2 ^a^	2.08 ± 0.14 ^a^	41.6 ± 0.5 ^a^	12.4 ± 0.2 ^b^
S/SP80:20	7.24 ± 0.05 ^a^	57.1 ± 0.1 ^b^	7.2 ± 0.1 ^a^	2.64 ± 0.28 ^a^	42.2 ± 0.8 ^a^	11.0 ± 0.2 ^a^
S80	7.88 ± 0.06 ^b^	55.3 ± 0.4 ^a^	7.0 ± 0.2 ^a^	2.08 ± 0.14 ^a^	41.6 ± 0.5 ^a^	12.4 ± 0.2 ^b^
S/WP80:20	6.02 ± 0.29 ^a^	58.2 ± 1.5 ^b^	6.9 ± 0.3 ^a^	1.97 ± 0.08 ^a^	44.5 ± 0.3 ^b^	10.0 ± 0.5 ^a^
S80	7.88 ± 0.06 ^b^	55.3 ± 0.4 ^a^	7.0 ± 0.2 ^b^	2.08 ± 0.14 ^a^	41.6 ± 0.5 ^a^	12.4 ± 0.2 ^b^
S/ALB80:20	5.68 ± 0.14 ^a^	58.2 ± 0.0 ^b^	6.4 ± 0.0 ^a^	2.42 ± 0.05 ^a^	44.9 ± 0.2 ^b^	10.2 ± 0.6 ^a^
S70	7.44 ± 0.53 ^a^	55.1 ± 0.3 ^a^	6.6 ± 0.0 ^a^	1.66 ± 0.12 ^a^	42.9 ± 0.7 ^a^	11.4 ± 0.4 ^b^
S/G70:30	6.63 ± 0.05 ^a^	56.5 ± 0.3 ^b^	6.9 ± 0.2 ^a^	1.25 ± 0.22 ^a^	45.0 ± 0.4 ^a^	9.5 ± 0.4 ^a^
S70	7.44 ± 0.53 ^a^	55.1 ± 0.3 ^a^	6.6 ± 0.0 ^a^	1.66 ± 0.12 ^a^	42.9 ± 0.7 ^a^	11.4 ± 0.4 ^a^
S/SP70:30	7.17 ± 0.68 ^a^	57.8 ± 0.7 ^b^	7.3 ± 0.0 ^b^	2.03 ± 0.02 ^b^	43.7 ± 0.0 ^a^	10.4 ± 0.0 ^a^
S70	7.44 ± 0.53 ^b^	55.1 ± 0.3 ^a^	6.6 ± 0.0 ^a^	1.66 ± 0.12 ^a^	42.9 ± 0.7 ^a^	11.4 ± 0.4 ^b^
S/WP70:30	5.38 ± 0.42 ^a^	57.1 ± 1.3 ^a^	6.8 ± 0.2 ^a^	2.58 ± 0.08 ^b^	44.9 ± 0.1 ^a^	9.5 ± 0.3 ^a^
S70	7.44 ± 0.53 ^b^	55.1 ± 0.3 ^a^	6.6 ± 0.0 ^b^	1.66 ± 0.12 ^a^	42.9 ± 0.7 ^a^	11.4 ± 0.4 ^a^
S/ALB70:30	5.58 ± 0.21 ^a^	59.1 ± 0.3 ^b^	6.1 ± 0.1 ^a^	2.12 ± 0.44 ^a^	43.6 ± 0.0 ^a^	9.6 ± 0.6 ^a^

**Table 5:** Percentages of increase (+) or decrease (−) of ΔH_g_ and ΔH_r_ by protein addition. Differences between T_O_ (ΔT_O_), T_Or_ (ΔT_Or_), PW_g_ (ΔPW_g_), and PW_r_ (ΔPW_r_) compared with starch control_._ Ns: no significant differences. S: starch, G: gluten, SP: soy protein, WP: whey protein, ALB: ovalbumin.

[Protein]	Effect	Protein	ΔH_g_ (%)	ΔT_O_ (°C)	ΔPW_g_ (°C)	ΔH_r_ (%)	ΔT_Or_ (°C)	ΔPW_r_ (°C)
**10%**	Protein addition	G	−10.8	Ns	Ns	Ns	Ns	Ns
		SP	Ns	Ns	Ns	Ns	+4.3	Ns
		WP	Ns	Ns	−1.1	Ns	+6.5	Ns
		ALB	−16.5	+2.5	−0.8	Ns	+7.6	−2.5
**20%**	Protein addition	G	−14.3	+1.0	Ns	Ns	Ns	−1.7
		SP	−8.2	+1.8	Ns	Ns	Ns	−1.2
		WP	−23.6	+2.8	Ns	Ns	+2.9	−2.5
		ALB	−27.9	+2.8	−0.6	ns	+3.3	−2.3
**30%**	Protein addition	G	Ns	+1.5	Ns	Ns	Ns	−1.9
		SP	Ns	+2.7	+0.7	+22.8	Ns	Ns
		WP	−27.7	Ns	Ns	+55.9	Ns	−1.9
		ALB	−24.9	+4.1	−0.5	Ns	Ns	Ns

## Data Availability

The original contributions presented in this study are included in the article/Supplementary Materials. Further inquiries can be directed to the corresponding author.

## Supplementary Materials

The following supporting information can be downloaded at: https://www.mdpi.com/article/10.3390/foods14223865/s1, Figure S1: Principal component analysis. All samples; Figure S2: Principal component analysis. Samples with 10% protein addition; Figure S3: Principal component analysis. Samples with 20% protein addition; Figure S4: Principal component analysis. Samples with 30% protein addition.
