# Supplementary material for: Effect of Food Proteins on Wheat Starch Pasting and Thermal Properties
Source: Foods. 2025 Nov 12;14(22):3865. doi: 10.3390/foods14223865 (PMC12651088; doi:10.3390/foods14223865)
Supplement: Supplementary file 1 [file foods-14-03865-s001.zip › foods-3961781-supplementary.pdf]

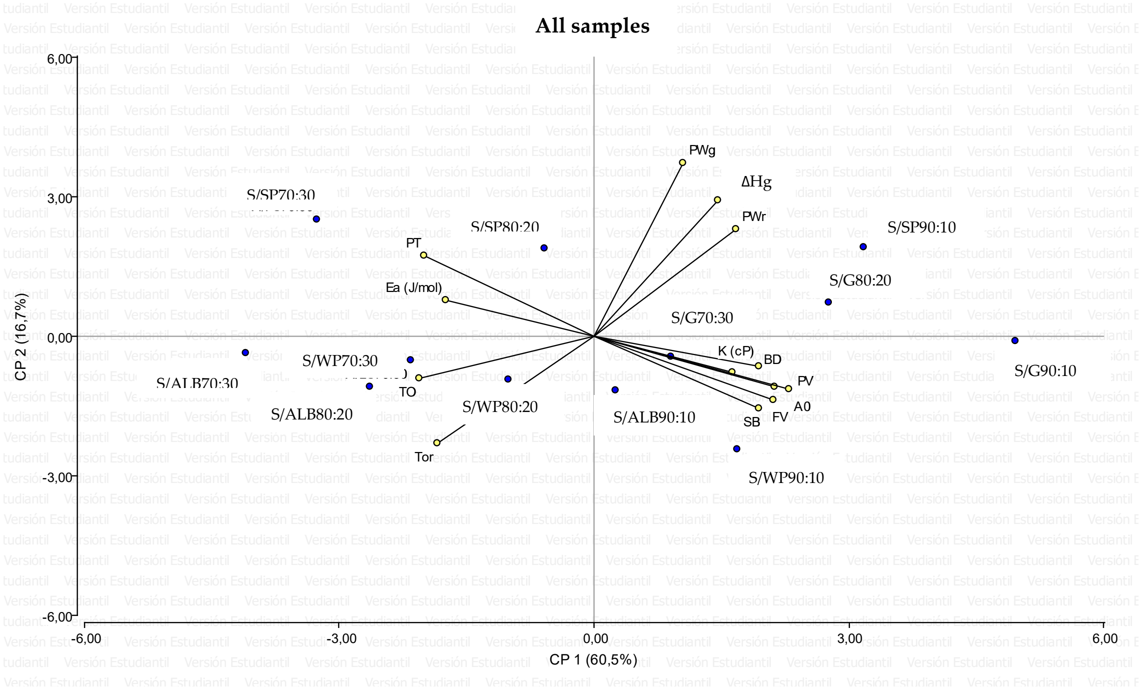

Figure S1. Principal component analysis. All samples.

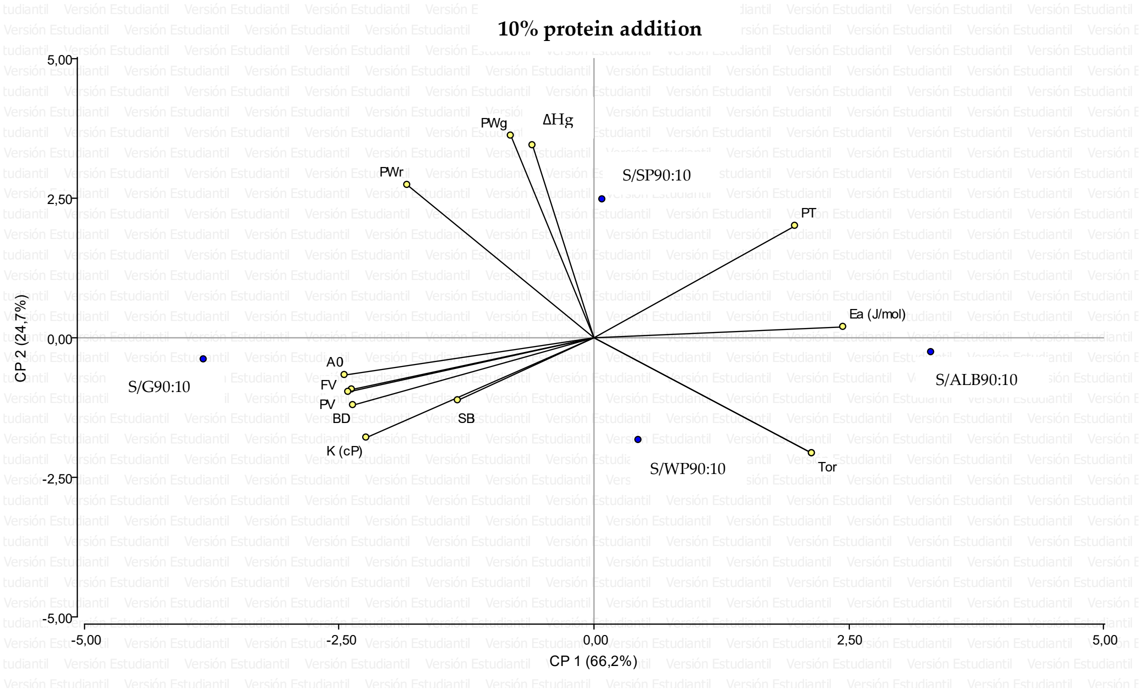

Figure S2. Principal component analysis. Samples with 10% protein addition.

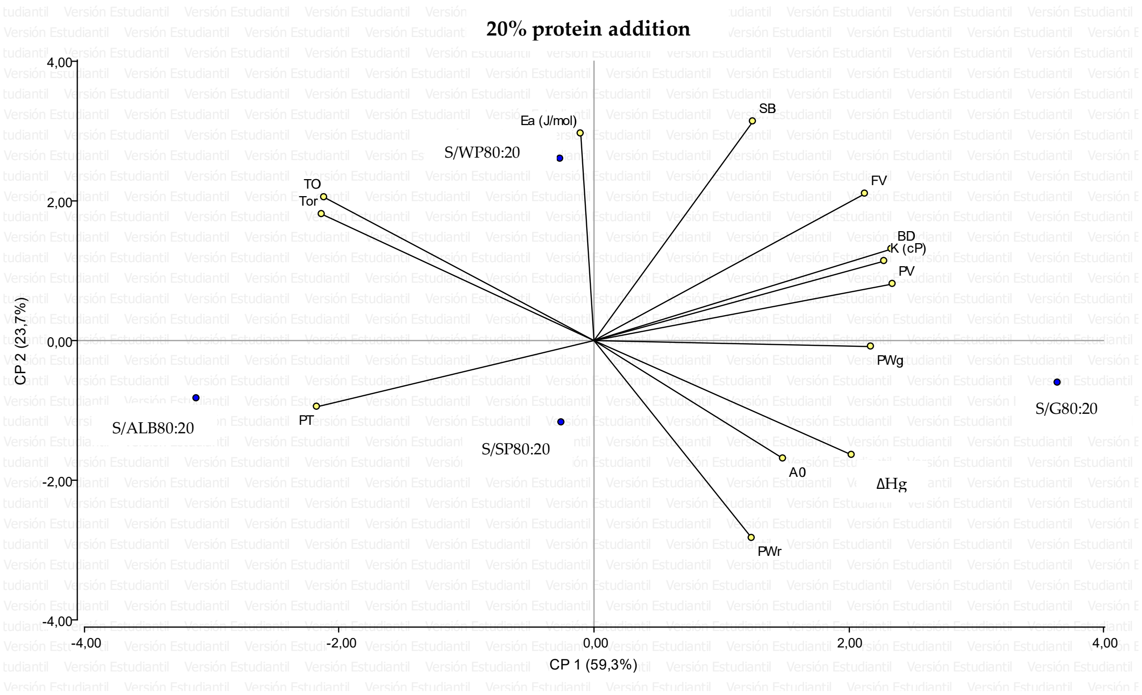

Figure S3. Principal component analysis. Samples with 20% protein addition.

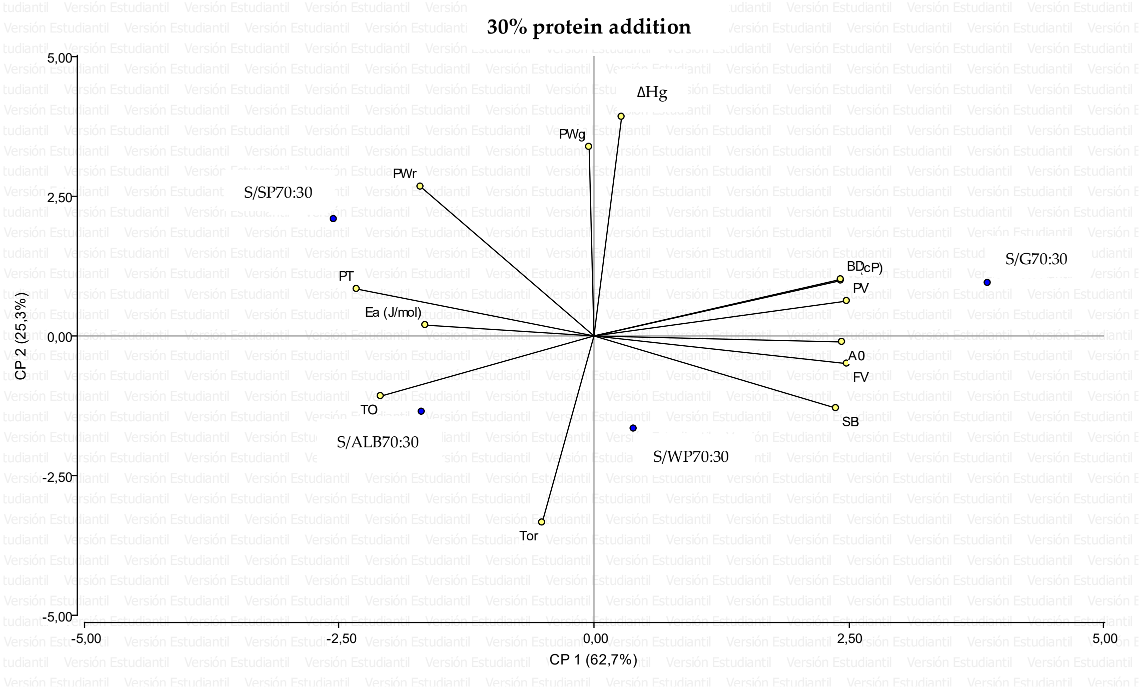

Figure S4. Principal component analysis. Samples with 30% protein addition.
